# Determinants of Outcome Among Critically Ill Police Personnel With COVID-19: A Retrospective Observational Study From Andhra Pradesh, India

**DOI:** 10.7759/cureus.20394

**Published:** 2021-12-13

**Authors:** Limalemla Jamir, Mukesh Tripathi, Sumita Shankar, Rakesh Kakkar, Ravishankar Ayyanar, Rajeev Aravindakshan

**Affiliations:** 1 Department of Community and Family Medicine, All India Institute of Medical Sciences (AIIMS), Mangalagiri, IND; 2 Department of Anesthesiology, All India Institute of Medical Sciences (AIIMS), Mangalagiri, IND; 3 Department of Plastic Surgery, Rangaraya Medical College, Kakinada, IND; 4 Law & Order, Andhra Pradesh Police Department, Mangalagiri, IND

**Keywords:** covid-19 mortality, covid-19, mechanical ventilation, remdesivir, povidone iodine, police personnel, prone positioning

## Abstract

Background and Aims: Police personnel have been key frontline workers throughout the coronavirus disease 2019 (COVID-19) pandemic. This study was conducted to assess the correlates and outcomes of critically ill police personnel.

Methods: This retrospective observational study analyzed key parameters of hospitalized police personnel who were critically ill with COVID-19 in Andhra Pradesh, India, between June and October 2020. Survival was analyzed for correlation with body mass index, ABO/Rh blood group, co-morbidities, treatment (oxygen therapy, prone positioning, mechanical ventilation, remdesivir, Ivermectin, oral and nasal topical povidone-iodine). We also performed Cox proportional hazard analysis with relevant function plots.

Results: The majority of the 266 patients were male (n = 259; 97.4%) and obese (75.2%). The overall mortality of patients was 38% (n = 101). COVID-19 mortality increased significantly with age (p = 0.019) and BMI (p = 0.030) in the bivariate analysis. There was no significant difference between blood group (p = 0.297), co-morbidity (p = 0.582) and COVID-19 outcome. Multivariable-adjusted hazard ratios (HRs) and 95% confidence intervals (CIs) of the risk factors for COVID-19 mortality were males (HR 4.89, 95% CI: 1.020-23.430) and ventilator therapy (HR 7.5, 95% CI: 4.527-12.296). The protective factors were symptom onset to reverse transcription polymerase chain reaction (RT-PCR) report interval (HR 0.36, 95% CI: 0.158-0.814), prone positioning (HR 0.43, 95% CI: 0.197-0.915), and use of povidone iodine (HR 0.43; 95% CI: 0.273-0.692).

Conclusion: COVID-19 mortality among critically ill hospitalized police personnel was reduced by time to diagnostic test result, prone positioning, and povidone-iodine use and increased with male gender and mechanical ventilation.

## Introduction

Police personnel have been key frontline workers along with healthcare personnel in the coronavirus disease 2019 (COVID-19) pandemic caused by the severe acute respiratory syndrome coronavirus 2 (SARS-CoV-2) virus. Law enforcement personnel are the backbone of maintaining public order while ensuring continuity of essential services during nationwide lockdowns and assisting with high-risk contact tracing of COVID-19 cases [1]. The frequent exposure of police personnel to SARS-CoV-2-contaminated environments has inadvertently caused acute stress and infection. Many of them have succumbed to COVID-19 during the pandemic [2-3].

Predictors of survival or mortality among critically ill COVID-19 patients have varied between studies. However, most reported that higher age, obesity, pre-existing respiratory diseases, hypertension, and diabetes mellitus indicate a poor prognosis. Conversely, intensive care interventions such as early awake prone positioning, mechanical ventilation, and investigational drugs such as remdesivir were reportedly protective against COVID-19 mortality [4,5]. Most health care facilities in India followed the national guidelines for the management of COVID-19 primarily based on pre-pandemic clinical expertise and early emerging evidence from across the world [6]. This study was conducted to assess outcomes and correlates in critically ill police personnel with COVID-19 in Andhra Pradesh, India.

## Materials and methods

Study design and setting

This was a retrospective observational study of critically ill police personnel with COVID-19, admitted to intensive care units across hospitals in the State of Andhra Pradesh, India, between June and October 2020.

The clinical records of critically ill adult patients (age 18 years and above) with COVID-19 as confirmed by reverse transcriptase-polymerase chain reaction (RT-PCR) testing, were examined. Those who had an event, either discharge (survivor) or death (non-survivor), were included in the study. Those who were undergoing treatment during the study period (i.e., without an event) were excluded.

Data collection and procedures

A pre-defined case record form was used to collect data for the study. Individual data of patients that fulfilled the inclusion criteria were recorded electronically or in paper format, retrospectively until October 2020. We collected information on age, gender, district, ABO and Rh blood group, history of contact with COVID-19 case with date if available, body mass index (BMI), co-morbidities, date of symptom onset, date of RT-PCR report, date of initiation and duration of oxygen therapy and mechanical ventilation, date of discharge or date when patient died. Data were obtained on treatment modalities such as oxygen therapy, awake prone positioning, mechanical ventilation, remdesivir, Ivermectin, and adjuvant therapy with povidone-iodine (nasal and oral topical application).

Due to the frequent contact of police personnel with COVID-19 cases in the State and the high risk of exposure to the virus, they had been provided with bottles of commercially available povidone-iodine gargle (2% solution) as a preventive and adjuvant measure against COVID-19.

The povidone-iodine solution was diluted with potable water (1:1) and applied intranasally in the anterior nares and to the nasal cavity walls as far as possible with cotton buds, followed by gargling for at least 30 seconds. This was to be done at least twice daily, before and after work hours, up to four times a day.

Operational definitions for the study

A case of COVID-19 was defined as laboratory RT-PCR confirmed SARS-CoV-2 infection from nasopharyngeal or oropharyngeal swab specimens from the patient.

For this study, critically ill patients were defined as those categorized as severe COVID-19 patients admitted to intensive care units (ICU).

The Asian body mass index cut-off levels (World Health Organization) were used for categorizing nutritional status. These are underweight (<18.5 kg/m2), normal BMI (18.5-22.9 kg/m2), overweight (23.0 -24.9 kg/m2) and obese (≥25 kg/m2).

Statistical analysis

Statistical analysis was done using Statistical Package for Social Sciences (SPSS) software (version 22.0; IBM Corp., Armonk, NY, USA). Microsoft Excel spreadsheet software was used to collate the information and calculate time intervals such as the time from symptom onset to test result and from symptom onset to death or discharge. Data were summarized as mean (standard deviation) and median (inter-quartile range) for quantitative data (Shapiro Wilk test < 0.05) and as frequency (percentages) for qualitative data. Bivariate analysis was done using the Pearson chi-square test or Fisher’s exact test and Mann-Whitney U test (non-parametric: duration data) for outcome as died versus survived (discharged). Cox regression analysis (univariable and multivariable) was done with COVID-19 mortality as the outcome variable. The adjusted analysis revealed a limited subset of variables that made a statistically significant (p < 0.05) contribution to mortality. Time-to-event curves were used to interpret the hazard ratios obtained by Cox proportional hazards regression. The patients were classified based on propensity variables that predicted the outcome, such as time to diagnostic test result (relatively milder cases being diagnosed late) and the use of a ventilator (severe cases requiring mechanical ventilation).

Ethics statement

The study was approved by the institutional ethics committee and complies with the Declaration of Helsinki. All India Institute of Medical Sciences (AIIMS), Mangalagiri, Andhra Pradesh issued approval IEC-AIIMS/Mangalagiri/2020-21/40. Informed consent was waived due to the retrospective nature of the study.

## Results

Characteristics of critically ill COVID-19 patients

A total of 266 critically ill COVID-19 patients were included in this study. This comprised 101 COVID-19 patients that passed away and 165 patients that recovered and were discharged from the health facilities (Table 1). The majority of the patients (97%) were male, and most (59.8%) were aged 50 years and above. COVID-19 mortality increased significantly with age- 2%, 30.7%, and 67.3%, for age groups 20 to 34 years, 35 to 49 years, and over 50 years, respectively (p = 0.019). The most common ABO/Rh blood group was O-positive. However, there was no significant difference between blood groups of the patients and outcome of COVID-19 illness (p = 0.297).

**Table 1:** Characteristics of critically ill COVID-19 patients #Multiple responses possible

Variable	Total (n=266)	Died (n=101)	Survived (n=165)		p value	
Age group in years (n, %)						
20 to 34	19 (7.1)	2 (2.0)	17 (10.3)		0.019	
35 to 49	88 (33.1)	31 (30.7)	57 (34.5)			
50 and above	159 (59.8)	68 (67.3)	91 (55.2)			
Sex (n, %)						
Male	259 (97.4)	98 (97)	161 (97.6)		0.787	
Female	7 (2.6)	3 (3.0)	4 (2.4)			
ABO/Rh blood Group (n, %)						
A+	52 (19.5)	22 (21.8)	30 (18.2)		0.297	
B+	76 (28.6)	27 (26.7)	49 (29.7)			
O+	109 (41.0)	37 (36.6)	72 (43.6)			
Others	29 (10.9)	15 (14.9)	14 (8.5)			
Body Mass Index (n, %)						
Underweight (<18.5 kg/m^2^)	2 (0.8)	0 (0)	2 (1.2)		0.030^$^	
Normal (18.5–22.9 kg/m^2^)	20 (7.5)	13 (12.9)	7 (4.2)			
Overweight (23.0 –24.9 kg/m^2^)	44 (16.5)	19 (18.8)	25 (15.2)			
Obese (≥25 kg/m^2^)	200 (75.2)	69 (68.3)	131 (79.4)			
Co-morbidity (n, %)						
Present	147 (55.2)	58 (57.4)	89 (53.9)		0.582	
Absent	119 (44.7)	43 (42.6)	76 (46.1)			
Type of co-morbidity^# ^(n, %)						
Hypertension	77 (28.9)	27 (26.7)	50 (30.3)		0.533	
Diabetes mellitus	100 (37.6)	34 (33.7)	66 (40.0)		0.300	
Respiratory diseases	14 (5.3)	9 (8.9)	5 (3.0)		0.037	
Other diseases	8 (3.0)	5 (5.0)	3 (1.8)		0.147	
Management						
Oxygen therapy (n, %)						
Yes	243 (91.4)	95 (94.1)	148 (89.7)	0.219		
No	23 (8.6)	6 (5.9)	17 (10.3)			
Prone positioning (n, %)						
Yes	30 (11.3)	11 (10.9)	19 (11.5)	0.876		
No	236 (88.7)	90 (89.1)	146 (88.5)			
Mechanical ventilator (n, %)						
Yes	68 (25.6)	67 (67.3)	1 (0.61)	<0.0001		
No	198 (74.4)	33 (32.7)	164 (99.39)			
Remdesivir (n, %)						
Yes	181 (68.0)	60 (59.4)	121 (73.3)	0.018		
No	85 (32.0)	41 (40.6)	44 (26.7)			
Ivermectin (n, %)						
Yes	76 (28.6)	32 (31.7)	44 (26.7)	0.379		
No	190 (71.4)	69 (68.3)	121 (73.3)			
Povidone iodine (n, %)						
Yes	163 (61.3)	39 (38.6)	124 (75.2)	<0.0001		
No	103 (38.7)	62 (61.4)	41 (24.8)			

Most (75.2%) of the patients were found to be obese and COVID-19 mortality increased significantly in the higher BMI categories (12.9% vs. 18.8% vs. 68.3%; p = 0.030). More than half (55.2%) of the patients had a co-morbidity. Although there was no significant association between overall presence or absence of co-morbidity and outcome (0.582), respiratory diseases (bronchial asthma, chronic obstructive pulmonary disease [COPD], tuberculosis) and/or in addition to pre-existing diseases, was significantly associated with the outcome (p = 0.037). There were statistically significant differences between usage/non-usage of mechanical ventilation (p<0.0001), remdesivir (p = 0.018), povidone iodine (p<0.0001) and COVID-19 outcomes among critically ill patients.

Duration of symptom onset, test report, intervention and outcome (died or survived) among critically ill COVID-19 patients

The median (interquartile range [IQR]) duration between symptom onset and receipt of a positive RT-PCR report (p = 0.0009), symptom onset and outcome (p < 0.0001), positive RT-PCR report and outcome (p < 0.0001), oxygen therapy and outcome (p < 0.0001), ventilator therapy and outcome (p = 0.002), differed significantly between patients who died and who survived (Table 2).

**Table 2:** Median duration (days) and outcomes among COVID-19 patients. *Data are presented as median (IQR) days   ^Mann-Whitney U test

Variable	Total*	Died*	Survived*	p value^^^
Symptom onset to test report (n=260)	2 (1, 3)	2 (1, 4)	1 (1, 2)	0.0009
Symptom onset to outcome (n=260)	14 (9, 19)	9 (5, 16)	16 (12, 21)	<0.0001
Test report to outcome (n=262)	12 (7, 17)	6 (2, 12)	14 (11, 19)	<0.0001
Oxygen therapy to outcome (n=240)	8 (5, 12)	5 (2.5, 8)	9 (6.5, 13)	<0.0001
Ventilator usage to outcome (n=64)	4 (2, 9.5)	3 (2, 7)	13 (9, 21)	0.0019

Predictors of COVID-19 mortality

**Figure 1:**
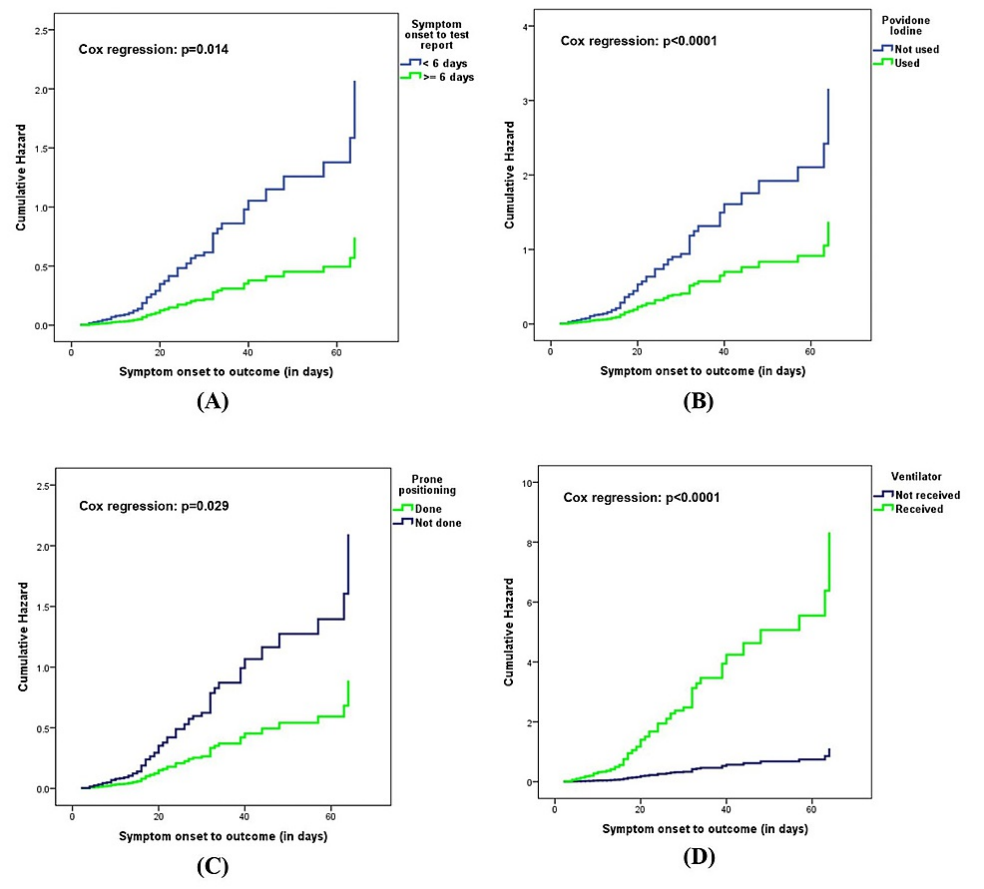
Hazard plots for symptom onset to test result (A); povidone iodine use (B); prone positioning (C) and ventilator use (D), with COVID-19 mortality as the outcome

As shown in Table 3 and Figure 1, hazard to fatal outcome further increased with time in the hospital when mechanical ventilation was done and prone positioning was not done. Addition of povidone-iodine to the care bundle significantly increased the probability of the patients recovering and being discharged from the hospital.

**Table 3:** Predictors of COVID-19 mortality, Cox regression analysis (adjusted) #CI, confidence interval; *p < 0.05; $6 days or more

Variable	Hazards ratio	95% CI^#^	p value
Age (years)			
20 to 34	Reference		
35 to 49	1.70	0.373–7.787	0.492
50 and above	1.66	0.377–7.348	0.501
Sex (male)	4.89	1.020–23.430	0.047^*^
Blood Group			
A+	Reference		
B+	1.17	0.616–2.229	0.631
O+	0.58	0.312–1.078	0.080
Other blood group	1.22	0.591–2.526	0.582
Body mass index			
Underweight/normal	Reference		
Overweight	1.07	0.465–2.452	0.880
Obese	1.06	0.550–2.028	0.871
Co-morbidity			
Hypertension	1.65	0.610–4.440	0.325
Diabetes mellitus	0.95	0.481–1.892	0.890
Respiratory diseases	2.98	0.864–10.279	0.083
Symptom onset to test result^$^	0.36	0.158–0.814	0.014^*^
Oxygen therapy	1.70	0.650–4.463	0.278
Prone positioning	0.43	0.197–0.915	0.029^*^
Mechanical ventilator	7.5	4.527–12.296	<0.0001^*^
Remdesivir	0.92	0.550–1.552	0.765
Ivermectin	1.53	0.883–2.665	0.129
Povidone iodine	0.43	0.273–0.692	<0.0001^*^

In the multivariable-adjusted Cox regression analysis, COVID-19 mortality was reduced by 57% (hazard ratio [HR] 0.43, 95% confidence interval [CI]: 0.197-0.915; p = 0.029) and 57% (HR 0.43; 95% CI: 0.273-0.692; p < 0.0001) if prone positioning was done and povidone-iodine was used, respectively. For patients with longer time to test report (six days or more), the risk of mortality was lower by 64% (HR 0.36, 95% CI: 0.158-0.814; p = 0.014), as compared to receiving test report within six days (early report for more severe cases). This was contrasted with the finding that COVID-19 mortality increased with male gender (HR 4.89, 95% CI: 1.020-23.430; p = 0.047) and mechanical ventilation (HR 7.5, 95% CI: 4.527-12.296; p < 0.0001), as compared to not using ventilator. Some other variables which were significant in the univariable Cox regression analysis such as age, overweight, co-morbidities lost significance in the adjusted analysis.

## Discussion

The present retrospective study showed that the independent risk factors of COVID-19 mortality among critically ill COVID-19 patients were being male and the use of a mechanical ventilator. The protective factors were a longer interval between symptom onset to test result (≥ six days), probably due to the prioritization of more serious cases for faster results, prone positioning and use of povidone-iodine as an oral gargle and topical nasal application.

Since most of the patients in the present study were male, a conclusive comparison cannot be made with the low number of female patients. However, previous studies have reported a poorer COVID-19 prognosis for male patients [7-8].

In the present study, ‘O’ Rh-positive was the most common ABO/Rh blood group and there was no statistically significant association of blood group with COVID-19 mortality. This contrasts with previous studies, which found ‘A’ Rh-positive to be the most common blood group among COVID-19 patients and increased the risk of severe COVID-19 [9-10]. Blood group lost its significance as a predictor of outcome among critically ill hospitalized COVID-19 patients amidst other determining factors in our analysis.

The high prevalence of overweight and obesity among COVID-19 patients in this study is in line with other studies [11,12]. However, the present study did not find obesity an independent predictor of COVID-19 mortality, unlike some other reports [13]. Although underweight COVID-19 patients have also been reported to have a poor prognosis in COVID-19, we could not analyze this due to the low number of underweight patients in our study. 

As reported earlier, high BMI, hypertension, diabetes mellitus, and respiratory diseases were prevalent among police personnel in the present study [14,15]. However, these co-morbidities were not independent predictors of COVID-19 mortality, which is in line with previous reports [5,16]. In contrast, chronic obstructive pulmonary disease has been reported to indicate a poorer prognosis in COVID-19 patients than asthma, obesity, hypertension, and diabetes [17,18].

Although we did not find oxygen therapy to be a predictor of COVID-19 outcome, its utility in preventing mortality and enabling ventilator availability has been reported [19]. We found, like other studies, a beneficial effect of prone positioning in reducing COVID-19 mortality and the intubation rate [20,21].

Similar to the present study, previous reports, including systematic reviews, have reported that mechanical ventilation, particularly if initiated early, substantially increased the risk of death [6]. However, early intubation in severe COVID-19 with acute respiratory distress syndrome reportedly improves survival rates [22]. The median duration of stay in the intensive care unit and the median duration of mechanical ventilation in the present study are also comparable to other studies [23,24]. Our findings of a significantly shorter duration between symptom onset or test report and outcome, shorter duration of oxygen therapy or mechanical ventilation in the deceased group of patients indicates rapid deterioration and highlights the need for vigilant prevention and control measures.

As reported by the World Health Organization’s global Solidarity Trial, which did not find that remdesivir reduced COVID-19 mortality, we did not find it independently influenced COVID-19 outcome in this study [25]. Another drug, ivermectin, used in COVID-19 management for its anti-inflammatory properties, was not found to independently predict COVID-19 mortality. Although studies have reported its utility in reducing clinical progression and mortality to some extent, its impact on critically ill COVID-19 patients could be sub-optimal [26].

We found that the use of povidone-iodine reduced COVID-19 mortality. This further confirms recent reports of the virucidal activity of povidone-iodine against SARS-CoV-2. It protects against clinical progression, secondary bacterial infections, and disease transmission [27,28]. Patients with uncontrolled diabetes mellitus are particularly vulnerable to COVID-19-associated mucormycosis, which is exacerbated by using steroids in COVID-19 treatment [29]. On the other hand, studies have proven the efficacy of topical povidone-iodine in otomycosis fungal infection [30]. The risk of mucormycosis in the present may have been low due to more judicious use of steroids in the initial phases of the COVID-19 pandemic as per government protocols or because the additional use of povidone-iodine prevented frank manifestations. Therefore, the effectiveness of povidone-iodine is speculated in our study. It may have averted worse outcomes had there been fungal infection, especially since a substantial number of the patients had diabetes and the comorbid conditions in the analysis proved to be non-significant in causing mortality.

To the best of our knowledge, this is the first reported study of outcome predictors among critically ill COVID-19 police personnel in India. The study therefore brought focus on this cadre of frontline workers and emphasizes the pressing need for strengthening healthcare services in this sector, not only during the COVID-19 pandemic but in the post-pandemic times as well. The findings are also comparable to those reported among other cadres of frontline workers and general population.

In this study, we were limited to observational data. However, we had the advantage of having prospective time-to-event data with several variables, which predicted the use of the initial agents in COVID-19 management. While symptom-specific treatment data, including the use of steroids was unavailable, the data provided useful insights into therapies that aided recovery. The adjustment of therapeutic variables, such as prone positioning and povidone-iodine, hastened recovery regardless of the background variables such as age, gender, and obesity which were earlier reported to worsen prognosis. Other limitations include data being self-reported such as date of symptom onset and test report date, which could not be confirmed. Other relevant clinical parameters and laboratory data were not available, hence a comprehensive analysis could not be done. Even though we adjusted for potential influencing factors, our results might still have been confounded by factors that were unavailable in the present study.

Conclusion

Our study found a significant association between age, BMI, and COVID-19 outcome among critically ill hospitalized police personnel. Independent predictors of COVID-19 mortality were male sex and the use of mechanical ventilation. Prone positioning and the use of povidone-iodine were protective factors against mortality. A longer interval (≥ six days) between symptom onset and test result is possibly an indirect indicator of relatively stable patients being deprioritized for reporting in high-volume, resource-limited COVID-19 laboratories.

## Conclusions

Our study found significant association between age, body mass index and COVID-19 outcome among critically ill hospitalized police personnel. Independent predictors of COVID-19 mortality were male patients and mechanical ventilation being risk factors whereas prone positioning and use of povidone-iodine were protective factors against mortality. Longer interval (≥ six days) between symptom onset and test result is possibly an indirect indicator of relatively stable patients being placed as second priority for reporting in high-volume, resource-limited COVID-19 laboratories.
